# Optimizing of a question prompt list to improve communication about the heart failure trajectory in patients, families, and health care professionals

**DOI:** 10.1186/s12904-020-00665-3

**Published:** 2020-10-15

**Authors:** Lisa Hjelmfors, Martje H. L. van der Wal, Maria Friedrichsen, Anna Milberg, Jan Mårtensson, Anna Sandgren, Anna Strömberg, Tiny Jaarsma

**Affiliations:** 1 Department of Health, Medicine and Caring Sciences, Division of Nursing Sciences and Reproductive Health, Linköping University, Linköping, Sweden; 2 Department of Health, Medicine and Caring Sciences, Linköping University, Linköping, Sweden; 3 Department of Cardiology, University Medical Center Groningen, University of Groningen, Groningen, The Netherlands; 4 Palliative Education & Research Centre, Vrinnevi hospital, Norrköping, Sweden; 5 Department of Advanced Palliative Home Care, Vrinnevi hospital, Norrköping, Sweden; 6 Department of Health, Medicine and Caring Sciences, Division of Prevention, Rehabilitation and Community Medicine, Linköping University, Linköping, Sweden; 7 Department of Nursing, School of Health and Welfare, Jönköping University, Jönköping, Sweden; 8 Center for Collaborative Palliative Care, Department of Health and Caring Sciences, Linnaeus University, Växjö, Sweden; 9 Department of Health, Medicine and Caring Sciences and Department of Cardiology, Linköping University, Linköping, Sweden

**Keywords:** Heart failure, Illness trajectory, End-of-life care, Communication, Question prompt list

## Abstract

**Background:**

The aim of this study was to optimize a Question Prompt List which is designed to improve communication about the heart failure trajectory among patients, family members, and health care professionals.

**Methods:**

Data were collected in a two-round Delphi survey and a cross-sectional survey, including patients with heart failure, their family members, and health care professionals working in heart failure care in Sweden and the Netherlands. Acceptability for and demand of the Question Prompt List were assessed.

**Results:**

A total of 96 patients, 63 family members and 26 health care professionals participated in the study. Regarding acceptability, most of the original questions were found to be relevant by the participants for inclusion in the Question Prompt List but some cultural differences exist, which resulted in two versions of the list: a Swedish version including 33 questions and a Dutch version including 38 questions. Concerning demand, participants reported that they were interested in discussing the questions in the revised Question Prompt List with a physician or a nurse. Few patients and family members reported that they were worried by the questions in the Question Prompt List and hence did not want to discuss the questions.

**Conclusions:**

This Question Prompt List has successfully been adapted into a Swedish version and a Dutch version and includes questions about the HF trajectory which patients, their families, and health care professionals perceived to be relevant for discussion in clinical practice. Overall, patients and family members were not worried about the content in the Question Prompt List and if used in accordance with patients’ and family members’ preferences, the Question Prompt List can help to improve communication about the heart failure trajectory.

## Background

Discussing the Heart Failure (HF) trajectory with patients and their families is recommended in clinical guidelines worldwide [1–3]. Health care professionals working in HF care should openly discuss any issue related to the illness trajectory, including prognosis, symptom management, options and preferences for palliation and end-of-life care as well as deactivation of devices with patients and their families. By doing so, the professionals help them to be prepared to live with a life-limiting illness and for the last phase of life [4, 5]. However, this open approach to communication about the HF trajectory is not widely implemented in clinical practice. Conversations in HF care mostly focus on disease management and less on patient preferences and goals for future care [6]. Discussing end-of-life care often becomes “the elephant in the room”, i.e. a topic which is rarely discussed as the patient waits for the professional to initiate the discussion, while professionals wait for a cue or a question from the patient to start such conversations [6]. At the same time, the amount of information about the HF trajectory wanted by the patient and the family member varies. Therefore, it is important for health care professionals to tailor their information according to the needs and preferences of each individual patient and family member [7, 8].

One way to improve communication about sensitive topics in clinical practice is by using a communication tool [9]. A tool that previously has been used in other chronic illnesses is a Question Prompt List (QPL): a prepared list of questions which is provided to the patient and a family member which enables them to identify those questions they wish to ask the health care professional [10, 11]. Studies involving QPLs report that patients and family members find such a tool useful to frame questions and prepare them for clinical visits and that they become more involved in the care and the decision-making process [10, 12] .

Previous research on QPLs are mostly from cancer care where such a tool has helped cancer patients and their families to give cues that they wished to discuss prognosis and end-of-life care with health care professionals [13]. Specially for cardiac patients, local or national information material is sometimes available, for example the “Difficult Conversations guide”, developed by the British Heart Foundation which supports health care professionals to talk about the HF trajectory and end-of-life care in a sensitive and supportive way with patients and their families [14]. A Dutch research project has recently identified characteristics of a tool to assess palliative care needs in HF patients and family members, and one important part of that tool is to facilitate conversations about palliative care needs [15]. Another example is the website heartfailurematters.org, an educational tool provided by the Heart Failure Association of the European Society of Cardiology that has a specific section which intends to help patients and families to discuss living with HF and plan for the end-of-life [16]. In other areas, checklists have been developed to assess palliative care needs of patients and family members [17, 18] however, conversation guides and similar materials that help patients and families to initiate discussions about the HF trajectory are scarce.

Recently, our research group including researchers from Sweden and the Netherlands, developed a QPL with the aim of improving communication about the HF trajectory in HF care. The first version of the QPL was developed in a co-design process [19] in Sweden and later on, translated to Dutch. In the co-design process, the concepts “Ideas groups” and “Prototyping” were used as described in the Health Service Co-design toolkit [20]. Patients with HF, their family members and health care professionals (physicians and nurses) from palliative and HF care participated in two ideas groups. In the first ideas groups, the participants suggested the need for a communication tool to improve communication about the HF trajectory, in the second ideas groups, the first prototype of the QPL was made, and the participants suggested useful questions and wording that could be included in the list. Following the second ideas group, the QPL was further refined by the researchers, based on the suggestions from the participants and relevant literature [21]. Finally, the revised QPL was sent to the participants in the ideas groups for a final evaluation. The QPL contained 45 questions grouped into 5 sections: 1) Heart failure and its impact on daily life, 2) Help and support when the illness deteriorates, 3) End-of-life, 4) Other questions family members may want to discuss, and 5) Other questions for patients with a heart failure pacemaker or an implantable cardioverter defibrillator [22].

To ensure the QPL is connected to clinical practice and has the potential to be used in the “real world”, further testing among patients, family members and health care professionals is needed. Accordingly, we know from previous studies in cancer care that QPLs developed in one country might need to be adapted for other countries depending on differences in how cultures approach sensitive topics [9]. Also, as there might be discrepancies in health care systems as well as cultural factors that may influence the content of the QPL [23], it is appropriate to tailor the QPL culturally [9], and include samples from both Sweden and the Netherlands in the testing.

The aim of this study was to optimize a Question Prompt List which is designed to improve communication about the heart failure trajectory among patients, family members, and health care professionals.

### Research questions

1. How do the participants perceive the title, the pictures, and the introductory text in the QPL?
2. Are the questions in the QPL relevant, are they clearly formulated, or do they need to be re-formulated?
3. Are there questions missing in the QPL?
4. Are the participants interested in discussing the questions in the QPL with a physician or a nurse?
5. What are the participants’ reasons for discussing/not discussing the questions in the QPL?

## Methods

### Design

**Fig. 1:**
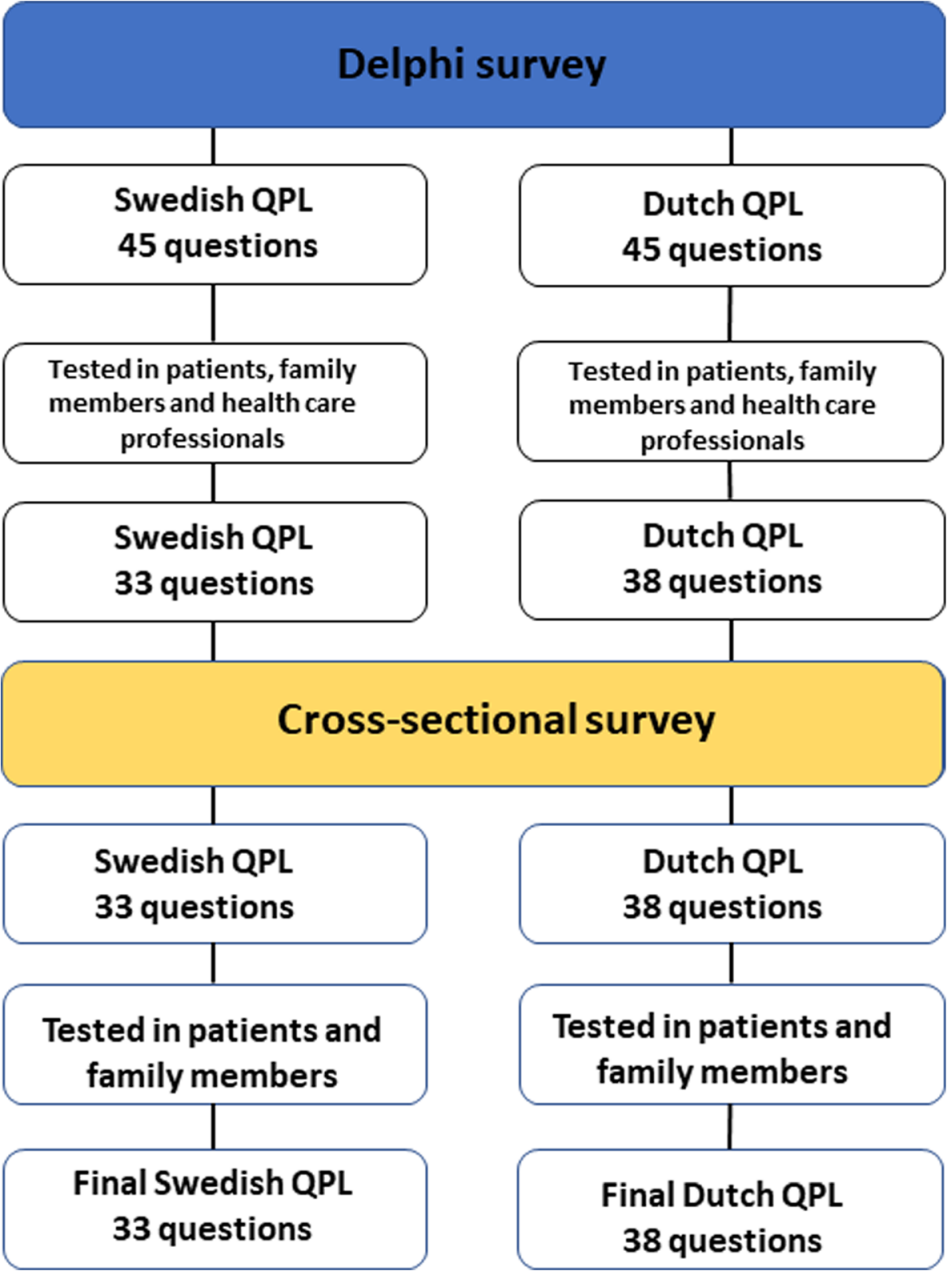
The process of testing acceptability of and demand for the Question Prompt List in Sweden and the Netherlands

This study was conducted in two steps (Fig. 1) with the first step using the Delphi technique [24] to explore acceptability [25] research questions 1–3, and the second step using a cross-sectional survey [26] to measure demand [25] research questions 4–5. In this study, acceptability focuses on individuals’ reactions to an intervention (the QPL) and demand addresses the estimated use of an intervention (the QPL) [24].

#### Study participants

Purposeful sampling [26] was used, and patients with HF were recruited by HF nurses at HF clinics in two county councils in Sweden and in two hospitals in the Netherlands. Patients were eligible if they were diagnosed with HF, class I-IV of the New York Heart Association Functional Classification, and had no cognitive impairment based on assessments of the recruiting nurses. Family members of patients were recruited when included patient consented to invite them. Health care professionals were recruited through the researchers’ professional networks and included nurses, physicians, social workers and psychologists working with HF patients.

#### Part 1: Delphi rounds

To report on the participants’ reactions to and perceived appropriateness of the QPL (acceptability), patients with HF, their family members and health care professionals in HF care in Sweden and the Netherlands were invited to take part in a two-round Delphi survey.

In the first round, participants were asked to comment on their reactions to the title, the pictures, and the introductory text in the QPL. They were asked to report on the relevance of each of the 45 questions in the QPL, using a Likert scale from 0 to 4 (0 = I do not know, 1 = very relevant, 2 = relevant, 3 = not so relevant, 4 = not relevant at all). They were also asked to report on the clarity (yes/no) and need for re-formulation of each question. Furthermore, they could suggest additional questions to be included in the QPL (Table 1).

**Table 1:** The Question Prompt List comprising the original 45 questions

**Nr**	**Section 1: Heart failure and its impact on daily life**
**1.**	What does heart failure entail?
**2.**	Is heart failure a serious illness?
**3.**	Is heart failure a lifelong illness?
**4.**	What can I do to improve my prognosis?
**5.**	What can I do to improve my condition?
**6.**	What is the likely impact of heart failure on my future?
**7.**	What goals are realistic for the future?
**8.**	What symptoms might I experience when my condition deteriorates in the future, and what should I do if they occur?
	**Section 2: Help and support when the illness deteriorates**
**9.**	Who can I talk to about things that worry or bother me?
**10.**	Who can my family talk to about things that worry or bother them?
**11.**	What treatment is available to me when I deteriorate?
**12.**	What support is available to me if I deteriorate and cannot look after myself?
**13.**	Can I choose where I want to be cared for when I deteriorate?
**14.**	Is it possible to be cared for at home when I deteriorate?
**15.**	What support is available to me if I choose to be cared for at home?
**16.**	Who can help me decide about my care?
**17.**	Who will have the overall responsibility for my care if I deteriorate?
	**Section 3: End-of-life**
	The questions below might not apply to your current situation. You do not need to read them if you do not want to, but you might want to discuss them in the future.
**18.**	Will someone tell me when I am approaching the end-of-life?
**19.**	Is it possible to predict how long someone has left to live?
**20.**	Will my heart failure prolong the end-of-life?
**21.**	How will I feel during my last days of life?
**22.**	What will happen to my heart failure treatment at the end-of-life?
**23.**	What happens in the body when you die from heart failure?
**24.**	Are breathing problems common at the end-of-life?
**25.**	Is pain common at the end-of-life?
**26.**	Is it common to experience anxiety at the end-of-life?
	**Section 4. Other questions family members may want to discuss**
**27.**	How do we agree on what the person who is ill can/should/is allowed to do?
**28.**	If needed, how do I get help to look after the person who is ill?
**29.**	How do I best help the person who is ill if they deteriorate?
**30.**	Who can I turn to if I have concerns about the care given to the person who is ill?
**31.**	Who can I talk to about things that worry and bother me?
	The questions below might not apply to your current situation. You do not need to read them if you do not want to, but you might want to discuss them in the future.
**32.**	What support is there for me when the illness deteriorates, and I feel that I cannot do anymore?
**33.**	How do I know that the end-of-life is approaching?
**34.**	What do I reply to the question” Am I going to die now?”?
**35.**	How do patients react when their illness deteriorates?
**36.**	How do family members react when the illness deteriorates?
**37.**	How do I know if the patient has died?
**38.**	What happens with the dead body?
**39.**	Who can help me organise the funeral?
	**Section 5. Other questions for patients with a heart failure pacemaker or an implantable cardioverter defibrillator**
**40.**	What will happen to my implantable cardioverter defibrillator (ICD) at the end-of-life?
**41.**	What impact will my heart failure pacemaker (CRT), pacemaker or implantable cardioverter defibrillator (ICD) have on my last days of life?
**42.**	What will happen to my ICD/CRT/pacemaker treatment at the end-of-life?
**43.**	How will my ICD be switched off?
**44**.	What will happen to my heart if my ICD is switched off?
**45.**	Can my ICD be switched off without mine or my family members’ knowledge?

*ICD* Implantable cardioverter defibrillator, *CRT* Cardiac Resynchronisation Therapy

A revised QPL was developed for the second round with 1) questions which the majority of the participants found relevant and thought should be kept on the list, 2) questions that were suggested for deletion because they were perceived as not relevant, and 3) questions that were added by participants. In the second round, participants were asked to state for each question whether the question should be kept, deleted or added. After this second round, a revised version of the QPL was made by the research group.

#### Part 2: Cross-sectional survey

To test the demand of the QPL, a cross-sectional survey was performed. A questionnaire including the revised questions in the QPL was sent out to a new sample of participants to assess the estimated use of the QPL [25]. The participants were asked to state if they wanted to discuss each of the questions in the QPL (yes/no/do not know) with a physician or a nurse ‘right now’ (i.e. at the time they were given the questionnaire), and report on the reason for their answers.

### Data analysis

Descriptive statistics were used to analyse the data in the study. The Delphi technique was used as a tool to receive feed-back on possible areas that could be optimised in the QPL. Round 1 questions that were perceived as not relevant by 25% or more of the participants were specifically discussed in the research group concerning formulations and content. Some of these questions were re-worded and added to round two for participants to comment on; some were suggested for deletion. Questions that were perceived as relevant by 50% or more of the participants in rounds 1- and 2 were kept in the QPL. The opinions of the participants had the biggest impact on the results, but the researchers also took on an active role in the analysis, contributing their knowledge to the optimizing of the QPL. The researchers’ role in the analysis of data concerned i. e. bringing in knowledge from relevant literature when designing the QPL, deciding on the ordering of the questions in the list as well as their final wording, using suggestions from all participants.

### Ethical considerations

Ethical approval was granted for this study by the Swedish Ethical Review Authority in Linköping (Dnr. 2017/464–32, 2018/409–32). The Medical Ethical Committee of the University Medical Centre Groningen (UMCG) concluded that no additional approval of the committee was needed (METC 2018/169). Written informed consent was obtained from all patients before commencement of the study.

Box 1 An example of one of the questions and the different response alternatives in the cross-sectional survey

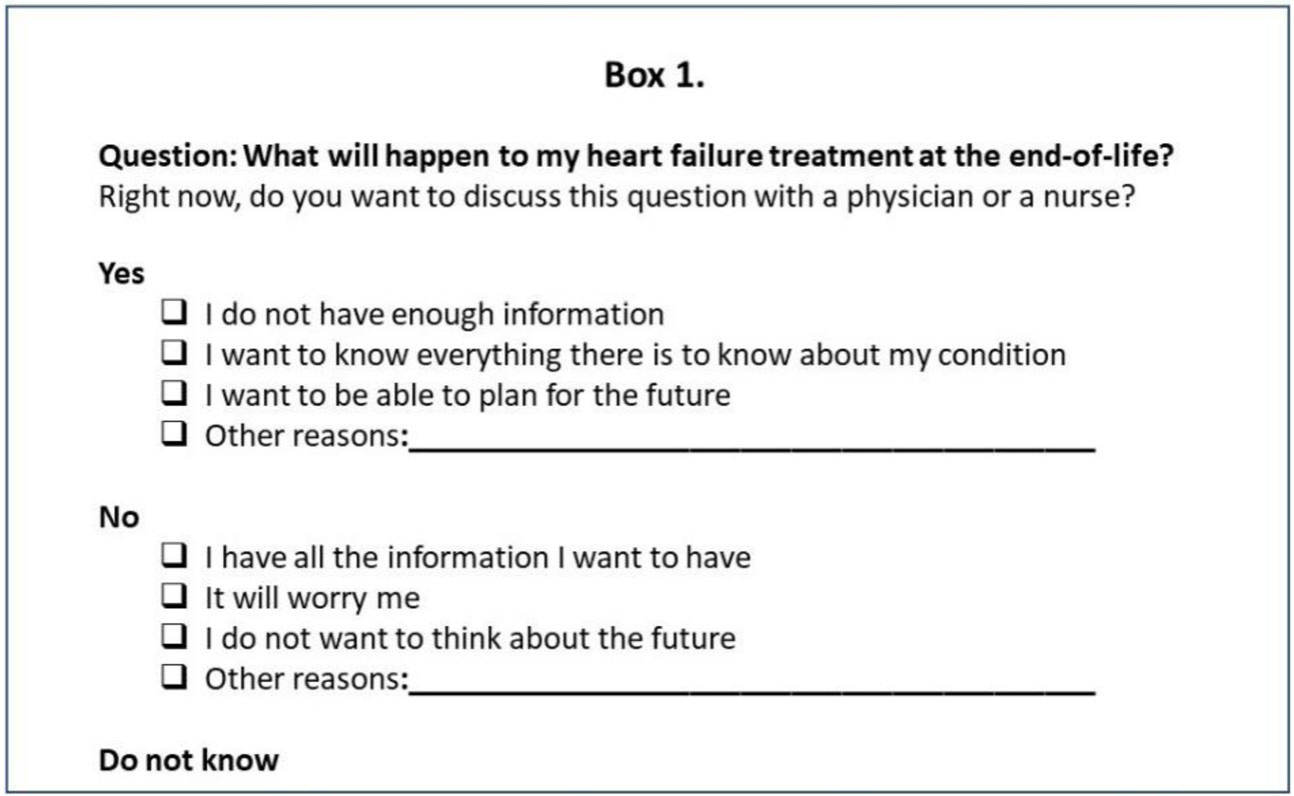

## Results

### Participants in the Delphi survey

A total of 71 participants were approached and 57 responded in the first round (17 patients, 14 family members and 26 professionals; response rate 79%). Patients had a mean age of 70 years (± 9), 78% were male and 89% were married or living with a partner. Most of the patients (56%) were in NYHA-class III and 72% of them had an ICD or CRT device. Family members had a mean age of 67 years (± 11), 71% were female and most of them (93%) were living together with the patient. Only one family member was the child of a patient. The professionals (11 HF nurses, 1 palliative care nurse, 8 cardiologists, 2 general practitioners, 1 geriatrician, 2 social workers and 1 psychologist) had a mean age of 43 years, 54% were female, and the majority of them (85%) worked at a hospital. In total, 4 patients, 6 family members and 5 health care professionals did not respond in the first round.

In the second round, 68 participants who responded in the first round were approached, and 51 participants responded (14 patients, 12 family members and 25 professionals, response rate 75%). In total, 5 patients, 6 family members and 6 health care professionals did not respond.

### Acceptability of title, pictures, and introductory text of the QPL

Overall, most of the participants in both samples endorsed the title, the two pictures and the introductory text of the QPL. Comments were made such as: the title could be shorter, and the picture on the front page (showing an older man and woman, smiling while talking to a health care professional) was found to be “too positive” with a tone that did not correspond with the serious content of the QPL. There were Dutch patients/family members who did not recognise themselves in the couple shown in the picture. There were also smaller changes suggested for the introductory text.

### Feedback on the questions in the QPL, Delphi rounds 1- and 2

**Fig. 2:**
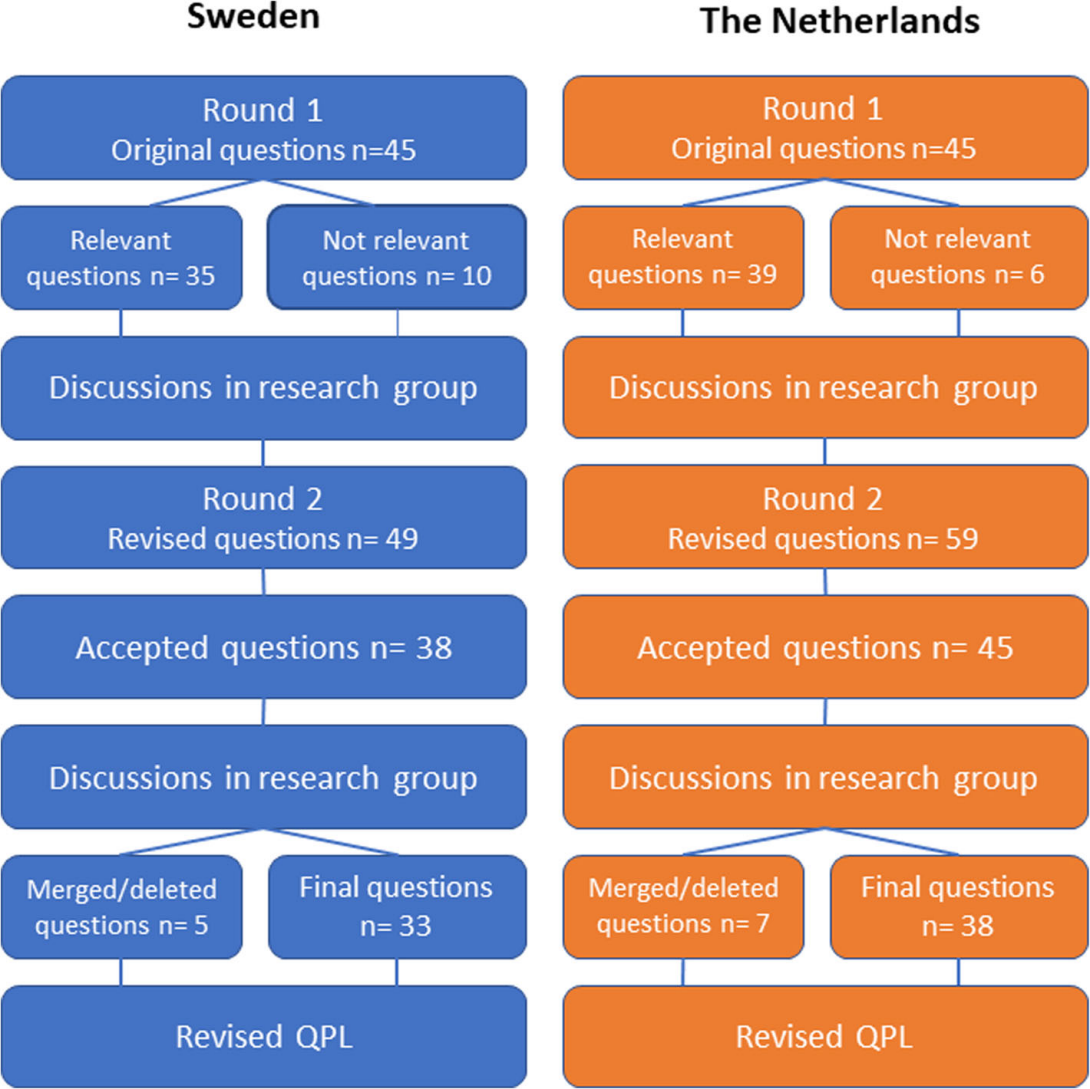
Results of the Delphi rounds in Sweden and the Netherlands. *QPL,* Question Prompt List

Forty-five of the questions in Sweden and thirty-nine in the Netherlands were perceived as relevant and clear by the participants, but for some of the questions, re-formulations were suggested to make them clearer (Fig. 2). Most of the questions that were perceived as not relevant concerned issues around end-of-life care. Participants showed concern regarding the inclusion of these questions in the QPL, describing that those questions could be too confronting, causing distress.

After Delphi round 1, the research groups in both countries discussed the results and discussed which questions should be kept, merged and/or re-formulated as they had similar content, which questions could be deleted from the QPL and also which new questions could be added that were found to be missing in the QPL.

In the second Delphi round, the Swedish QPL included 49 questions of which the researchers suggested that 33 should be kept, 12 questions deleted, and 4 new questions added. In the second Delphi round in the Netherlands, the QPL included 59 questions of which the researchers suggested that 33 should be kept, 12 questions deleted, and 12 new questions added. The participants were asked to state if they agreed on the suggestions.

In round 2, the participants agreed that 38 proposed questions in Sweden and 45 questions in the Netherlands should be kept in the list. In the research group discussions, some questions with similar content were merged or deleted which resulted in a final QPL for Sweden including 33 questions and 38 questions for the Dutch QPL. Based on the participants’ answers questions that that were deleted were for example “is it possible to predict how long someone has left to live?”, “what happens in the body when you die from HF?”, and “how do I reply to the question “Am I going to die now?”.

### Participants in the cross-sectional survey

A total of 115 patients (49 in Sweden, 66 in the Netherlands, Table 2) were approached; 29 Swedish and 50 Dutch patients responded to the questionnaire. Eight Swedish and 41 Dutch family members of these patients also responded to the questionnaire (Table 3). Mean age of the patients was 76 years, 38% were female and most of them (63%) were in NYHA-class III. Family members were most often partners and were younger then the patients (mean age 66 years).

**Table 2:**
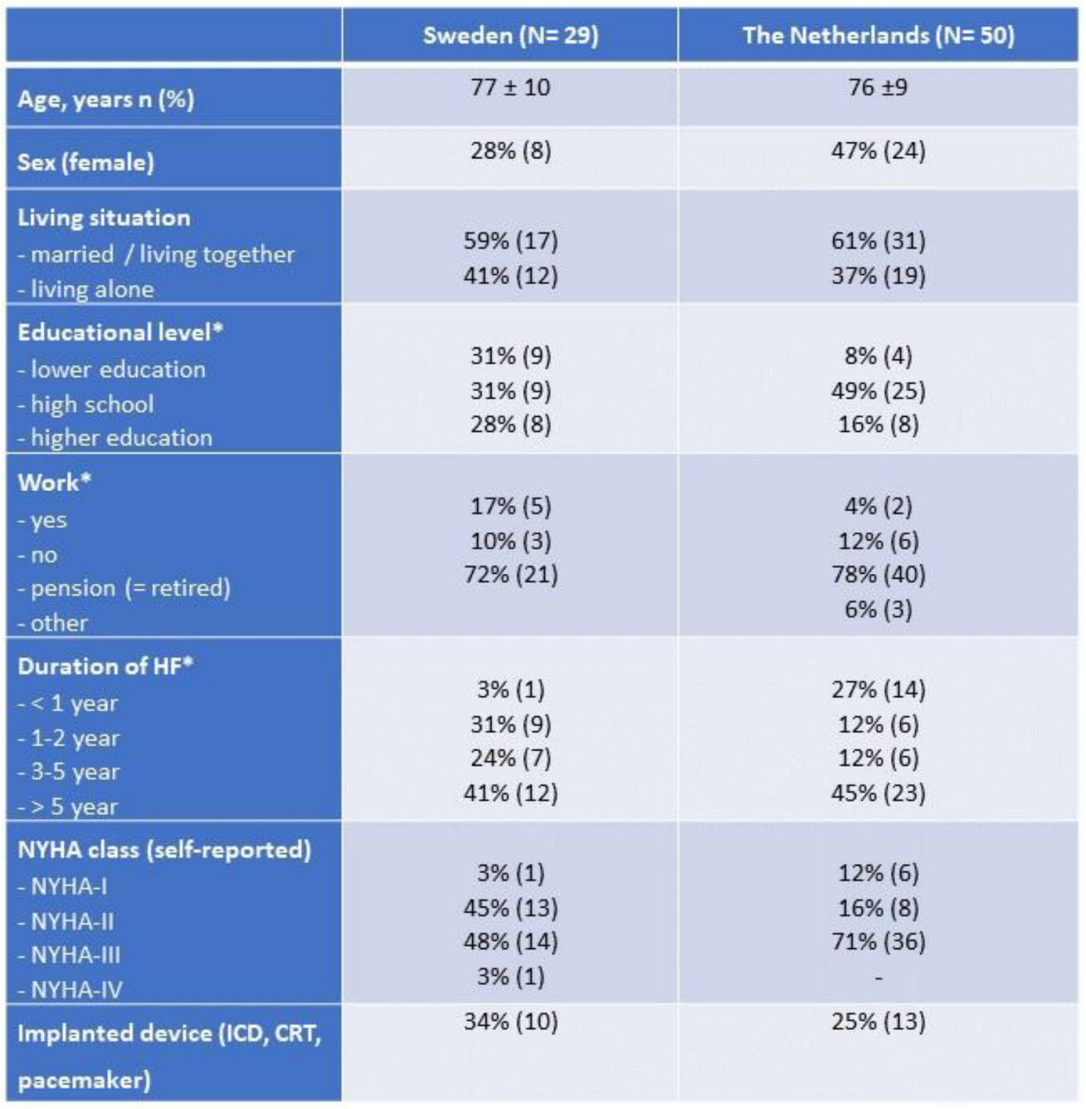
Baseline characteristics of patients in the cross-sectional survey in Sweden and the Netherlands

*HF* Heart Failure, *NYHA* New York Heart Association Functional Classification, *ICD* Implantable cardioverter defibrillator, *CRT* Cardiac Resynchronization Therapy. *These items have missing data

**Table 3:**
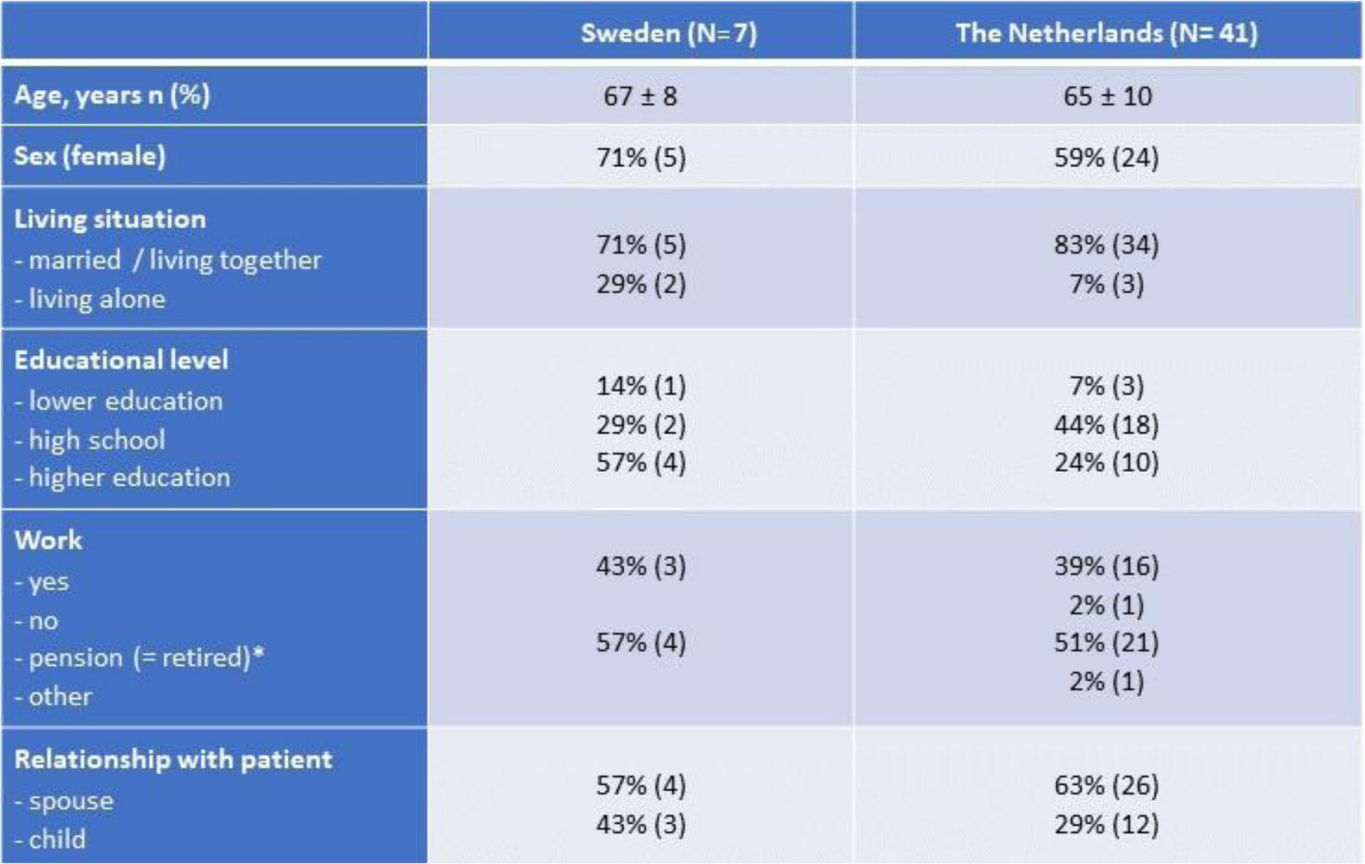
Baseline characteristics of family members in the cross-sectional survey in Sweden and the Netherlands

*This item has 5% missing data

### Demand of QPL- the cross-sectional survey

For each question in the QPL, at least 25% of the participants reported that they were interested to discuss the question with a physician or a nurse. Overall, the most reported reason why participants wanted to discuss questions in the QPL was that they wanted to know everything about the condition; seldomly they reported a need of information to be able to plan for the future (maximum 3 patients in the Netherlands and 6 patients in Sweden per question and max 1 family member).

If participants did not want to discuss a question, they most often gave the reason that they already had enough information. If participants reported they did not want to discuss a question because they would be worried or they did not want to think about the future, it was often the same participants reporting this answer on more than one question.

#### Section 1- heart failure in daily life (6 questions in Sweden, 10 questions in the Netherlands)

**Fig. 3:**
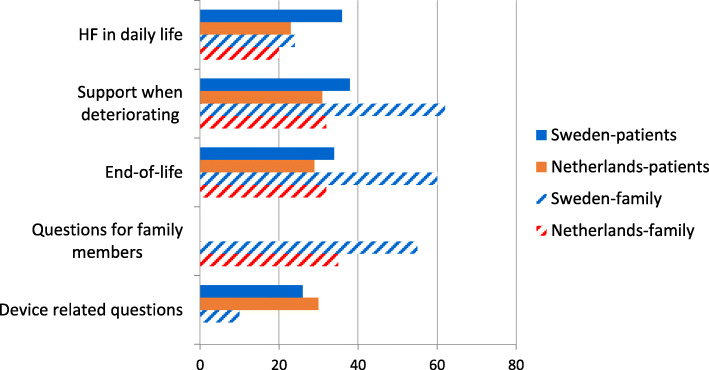
The mean percentage of how many patients/family members reported that they wanted (yes) to discuss the questions in the different sections of the Question Prompt List. No data was collected in Dutch family members concerning devices. *HF,* Heart Failure

In this section (Fig. 3), each question was selected for discussion by 31–41% of the patients in Sweden and by 18–33% of the patients in the Netherlands (average 36 and 23%, see Fig. 3). In both countries the question “What is the likely impact of HF on my future (Sweden)/what are the consequences of HF for my future”?(the Netherlands), was selected by most patients to be discussed (38 and 27%). All questions in this section were also reported by family members as relevant for discussion. In total, 4 of 29 patients in Sweden and 2 of 50 in the Netherlands did not want to discuss several questions in this section, either because they would be worried, or they did not want to think about the future. For example, the question ‘Is heart failure a serious illness?’ would worry 4 patients in Sweden and 2 in the Netherlands.

#### Section 2- support when deteriorating (9 questions in Sweden, 11 questions in the Netherlands)

In this section (Fig. 3), each question was selected for discussion by 28–45% of patients in Sweden and by 24–45% by patients in the Netherlands, with similar percentages reported by family members. The question that was most often selected for discussion in Sweden was “Can I choose where to be cared for if I deteriorate?” (45%) and in the Netherlands “What support is available to me if I choose to be cared for at home?” (46%). Family members also reported that they wanted to discuss these questions. In this section, 3 of 29 patients in Sweden and 5 of 50 patients in the Netherlands did not want to talk about several questions as they did not want to think about the future or because they would be worried. Five Dutch patients did not want to discuss the question “Which symptoms can occur when deteriorating?” because they did not want to think about the future and 3 Swedish patients did not want to discuss the question ‘Who can help to decide about my care?’ because they did not want to think about the future.

#### Section 3- end-of-life (4 questions in Sweden, 5 questions in the Netherlands)

In this section (Fig. 3), each question was selected for discussion by 28–38% of patients in Sweden and by 24–37% of patients in the Netherlands, with similar percentages reported by family members. Two questions were most often selected to be discussed by patients and family members in Sweden: “What will happen to my heart failure treatment at the end-of-life?” and “How will the last days in my life be, dying from heart failure, is there much suffering for example breathing problems and anxiety?”. One question was most often selected for discussion by patients and family members in the Netherlands: “What symptoms can occur in the last phase of my life?”. In this section, some patients did not want to talk about several questions as they did not want to think about the future,(6 of 29 patients in Sweden and 4 of 50 patients in the Netherlands), or because they would be worried. The question related to how they would know that the end-of-life was approaching was preferred not to be discussed by 5 Swedish and 4 Dutch patients because they did not want to think about the future.

#### Section 4- questions for family members (9 questions in Sweden, 7 questions in the Netherlands)

In this section (Fig. 3), only the answers from the family members are reported. All questions were selected for discussion by 29–71% of the family members in Sweden and by 29–41% of the family members in the Netherlands. The question “How do I best help my family member with heart failure if he/she deteriorates?” was selected most often by family members in both countries. In this section, only 1 of the 41 family members in the Netherlands reported that he/she did not want to talk about several questions in this section as he/she did not want to think about the future. Nobody selected that they would be worried talking about questions in this section.

#### Section 5- device-related questions (5 questions in Sweden, 5 questions in the Netherlands)

In this section (Fig. 3), only the answers from patients who reported they had a device and their family members (10 patients in Sweden and 13 patients in the Netherlands) were analysed. No family members in the Netherlands responded to this section. All questions in this section were selected for discussion by at least one of the participants. The question patients in Sweden and the Netherlands most often wanted to discuss (40%) was “What will happen when my ICD is switched off?”. Three Swedish patients responded that they did not want to talk about the question “What impact will my heart failure pacemaker (CRT), pacemaker or implantable cardioverter defibrillator (ICD) have on my last days of life?” because they would be worried.

#### Additional feedback from participants

Patients and family members in both countries provided additional comments to describe their reasons for why they did or did not want to discuss certain questions in the QPL at this moment. For some it seemed too early to discuss several questions at this point in the HF trajectory but later, it could be important to get more information. Some patients expressed how they wanted to live ‘in the here and now’, not worrying about possible deterioration in the future. On the other hand, participants also commented that it could be too late to have this kind of conversation, if the patient, for example, was very old.

## Discussion

This study describes the optimizing of a QPL to improve communication about the HF trajectory in HF care. The QPL was previously developed in a co-design process [19] together with the future users (patients/families and health care professionals) as it is known that for QPLs to impact communication positively, they need to be designed specifically for the intended population [27]. There is to our knowledge, no previous QPL developed for discussing the HF trajectory. In one study, Martinali et al. describe a checklist which aimed to prepare cardiac patients for a visit to the cardiologist [28]. The checklist included questions about medication, available treatments, risk factors and lifestyle, but few questions in that checklist were related to the HF trajectory. The QPL tested in our study focuses specifically on questions about the HF trajectory and end-of-life issues and is supposed to be used in discussions among patients, their families, and several health care professionals, not only by a cardiologist in physician- patient communication. Results from a systematic review in cancer care indicated that QPL is often one component of a complex intervention. When including a QPL it seems to increase patient participation, decrease psychological distress and improve recall of given information [23].

### Acceptability of QPL

The acceptability testing [25] focused on participants’ reactions and perceived appropriateness of the QPL. Based on participants’ suggestions, changes were made to the title, the pictures, and the introductory text of the QPL. Concerns from participants in the Delphi rounds (patients/families/professionals) regarding the inclusion of end-of-life questions in the QPL were considered. However, as the main aim with the QPL is to improve discussions about the whole HF trajectory, from prognosis to the last days of life, we decided to keep questions about end-of-life issues in the QPL but we re-formulated the questions, to try to make the tone less confronting while retaining the meaning of the questions. An alternative procedure to deal with the issue of end-of-life questions is described by Ekberg et al. who split their paediatric palliative care QPL into two version to allow end-of-life question to be provided separately [11]. For this QPL, it could mean having one version of the QPL for early referral and another version would address end-of-life questions. However, overall, patients, family members and health care professional in our study found questions related to the end-of-life appropriate for inclusion in the QPL and in the cross-sectional study all questions were selected for discussion.

Our findings show that patients and families in our study were not upset by the idea of discussing the more sensitive questions concerning the HF trajectory. Health care professionals, such as physicians, nurses and other relevant professionals, need to discuss the HF trajectory with patients in order to improve their understanding of their illness and address possible care needs of patients and their families [1, 2, 29]. However, health care professionals may be afraid they would diminish hope for the future or make patients and families worried if they were to discuss the HF trajectory in clinical practice [30–33].

A QPL needs to be used with care as patients have different approaches to cope with HF and also want different amounts of information depending on their current situation [7]. Some patients may only want to face part of the reality as a way of maintaining hope [34]. Therefore, we suggest that professionals should openly discuss any issue related to the HF trajectory with patients and their families and always strive to maintain a positive future orientation, hoping for better moments or for a good death, in accordance with the patient’s own values and beliefs [35]. In that way, the professionals can help them to keep a hope for the future in that sense that they will get help to prepare for living as good a life as possible, despite living with a life-limiting illness [5, 36].

### Cultural aspects in communication

We found in our acceptability testing of the QPL that most of the original 45 questions were perceived as appropriate by the participants in both Sweden and the Netherlands and there were no huge differences in the participants’ opinions. Nevertheless, some cultural discrepancies exist in the content and in the amount of questions, resulting in two versions of the QPL: a Swedish version including 33 questions and a Dutch version including 38 questions. The Dutch QPL included more questions in the sections “Heart Failure in daily life”, “Support when deteriorating”, and “End-of-life”. The Swedish QPL included more questions in the section covering “Questions for family members”.

We suggest that the questions in the Swedish and the Dutch QPLs can function as a blue-print for others to use. These QPLs can provide possible questions to be included in future QPLs, but we recommend that cultural adaptation of the questions should be done to fit the intended culture optimally and also provide the opportunity for each country to add additional questions that might be missing in the original QPL [37]. Additional files show the final Swedish and Dutch questions in more detail (see Additional file 1) and the questions that were suggested to be deleted or added in the Swedish and Dutch Delphi rounds (see Additional file 2).

### Demand of the QP

The participants’ interest in using the QPL in clinical practice was assessed, and participants in both Sweden and the Netherlands reported that they were interested to discuss questions from all the five sections in the QPL. Some participants, particularly those who felt they lacked information, commented that the QPL could help them to select questions and initiate a discussion. This is in line with previous research concluding that providing a QPL in clinical practice can increase the number of questions a patient asks if the QPL is tailored to the patient’s needs [10, 23, 38]. In this study, the main reason why participants wanted to discuss questions in the QPL was that they wanted more information in general. We had expected a larger number of patients and family members to answer that they wanted to discuss the HF trajectory in order to plan for the future, as HF is a chronic illness with often a bad prognosis.

Importantly, few patients and family members reported that they were worried by the questions in the QPL and hence did not want to discuss the questions. This needs to be considered in clinical practice since one commonly reported barrier from health care professionals why they avoid conversations about the HF trajectory is the belief that patients might become worried if they initiate discussions about prognosis or end-of-life issues [6, 32].

For participants who did not want to discuss the questions in the QPL, the most reported reason was because they already had all the information that they wanted about the HF trajectory. Interestingly, all patients included in this study were visiting a HF clinic in Sweden or the Netherlands, where they were provided with dedicated time with professionals specialised in HF care. Nevertheless, around 30% of the participants declared a need for more information about the HF trajectory, indicating that we can do better in providing this kind of information to patients and their families.

We found in the open-ended answers from patients and family members that the timepoint at which they wanted to discuss certain questions in the certain sections in the QPL differed. Some participants wanted to discuss, for example end-of-life issues later in the HF trajectory, and perceived that it was too early at the time the questionnaire was administered. Hence, we suggest that there must be repeated opportunities for discussion when the patient and family members want more information. These repeated opportunities for discussion are also important as we know that many HF patients are not aware of their poor prognosis or the severity of HF [39, 40]. Many HF patients express a need to be better informed with adequate information [41] provided by professionals with honesty and competence [42]. The delivered information should still always be relevant to each HF patient’s actual situation [43], therefore a QPL can provide a useful tool, giving patients and family members a way of initiating a discussion, asking questions that are relevant to them in their current situation [10, 12, 44], and also “planting the seed” for questions that might be relevant to discuss in the future [45]. Hence, the health care professionals have a role in initiating a discussion about the HF trajectory and providing the QPL to patients and their families throughout the whole HF trajectory to make sure their information needs are satisfied.

### Family members’ need for information

Family members reported that they would like to discuss questions in all the sections of the QPL; sometimes they reported even higher needs for information than the patients did. Information needs may change between patients and their family members during the HF trajectory, whereas patients tend to want less information the closer they are to the end-of-life [46], but the family members instead have higher needs for information [43]. It may be suggested that two different versions of the QPL could be offered, one intended for the patient and another for the family members [11]. On the other hand, it is known that patients consider it as important that a family member is present in conversations with health care professionals [47], which is why we recommend use of the QPL to be in its current format, including questions for both patients and family.

### Strengths and limitations

Study participants were recruited from regions which are seen as average regions for each country. They consisted of a diverse group of patients, their families and health care professionals from Sweden and the Netherlands. Although our study population was selected from HF clinics, we still realise that participants were approached to be in a research study. Maybe only patients, families, and health care professionals with an open approach to discussing the HF trajectory were represented in the samples, but as we included four different samples from two different countries, we believe this risk is minimized.

The research team, involving 6 nurses, 1 physician and 1 behavioral scientist, provided their clinical experience and research knowledge of HF- and palliative care to inform the planning and performance of the study.

In the cross-sectional survey, the participants were asked if they would like to discuss the questions in the QPL with a physician or a nurse, as these are health care professionals that they in general meet the most. Additionally, we argue that the QPL is suitable for use by several other health care professionals as well, such as social workers, and psychologists. Accordingly, in the cross-sectional survey the participants were asked if they wanted to discuss the questions in the QPL “right now”. This may have affected the results and the final questions in the QPL.

There were some differences between the countries in sample sizes and NYHA class in the cross-sectional study. The Dutch samples included more patients and family members compared to the Swedish samples and a bigger proportion of the Dutch patients had more symptomatic patients in NYHA class III.

One possible limitation in data analyses in the cross-sectional survey was the use of predetermined answering options in the questionnaire. If only open answers would have been allowed it could have produced a more rich data, possibly providing more important insights into the reasons why or why not patients and their family members wanted to discuss the different questions in the QPL.

## Conclusions

The QPL, designed for communication about the HF trajectory, has successfully been adapted into one Swedish version and one Dutch version. It includes questions that patients, their families, and health care professionals perceived as relevant to discuss with health care professionals about the HF trajectory. Overall, patients and family members were not worried about the content in the QPL and if used in accordance with patients’ and family members’ preferences, the QPL can help to improve communication about the heart failure trajectory. The QPL is ready to be used in clinical HF care in Sweden and the Netherlands. Further research is needed to examine the optimal way of delivery of the QPL into clinical practice and to examine how patients/family/professionals experience using the QPL in daily practice.

## Supplementary information

**Additional file 1.** The final questions in the two versions of the QPL. SW, Sweden, NL, The Netherlands.

**Additional file 2.** The questions that were suggested to be deleted or added in the Swedish and Dutch Delphi rounds. SW, Sweden, NL, The Netherlands.

## Data Availability

The datasets used and/or analysed during the current study are available from the corresponding author on reasonable request.

## Abbreviations

CRT: Cardiac Resynchronisation Therapy
ICD: Implantable Cardioverter Defibrillator
HF: Heart Failure
NYHA class: New York Heart Association functional classification
NL: the Netherlands
QPL: Question Prompt List
SW: Sweden
