# Supplementary material for: Optimizing of a question prompt list to improve communication about the heart failure trajectory in patients, families, and health care professionals
Source: BMC Palliat Care. 2020 Oct 15;19:161. doi: 10.1186/s12904-020-00665-3 (PMC7566035; doi:10.1186/s12904-020-00665-3)
Supplement: Supplementary file 1 — Additional file 1. The final questions in the two versions of the QPL. SW, Sweden, NL, The Netherlands. [file 12904_2020_665_MOESM1_ESM.docx]

**Additional file 1.** The final questions in the two versions of the QPL. SW, Sweden, NL, The Netherlands.

| **SW** | **NL** | **Section 1: Heart failure and its impact on daily life** |
| --- | --- | --- |
| **1.** | **1.** | What does heart failure entail? |
| **2.** |  | Is heart failure a serious illness? (SW) |
|  | **2.** | How serious is it to have heart failure? (NL) |
| **3.** |  | Is heart failure a lifelong illness? (SW) |
|  | **3.** | Can heart failure be cured? (NL) |
| **4.** |  | What is the likely impact of heart failure on my future? (SW) |
|  | **4.** | What can I do to improve the course of the disease? (NL) |
| **5.** |  | What can I do to improve my prognosis and my condition? (SW) |
|  | **5.** | What can I do to improve my condition? (NL) |
|  | **6.** | What are the consequences of heart failure for my future? (NL) |
| **6.** | **7.** | What goals are realistic for the future? |
|  | **8.** | What can heart failure mean for the relationship with partner? (NL) |
|  | **9.** | Which phases of heart failure are there and in which phase am I? (NL) |
|  | **10.** | With whom can I talk when I have problems with coping with the disease? (NL) |
| **SW** | **NL** | **Section 2: Help, support and treatment when deteriorating** |
| **7.** | **11.** | What symptoms might I experience if my condition deteriorates in de future, and what should I do if they occur? |
| **8.** |  | What support and treatment are available to me if my illness deteriorates? (SW) |
| **9.** | **12.** | Who can I talk to about things that worry or bother me? |
| **10.** | **13.** | Who can my family talk to about things that worry or bother them? |
|  | **14.** | Which treatment can I get if I deteriorate? (NL) |
|  | **15.** | What support is available if I deteriorate and cannot care for myself anymore? (NL) |
| **11.** | **16.** | Can I choose where to be cared for if I deteriorate? |
| **12.** |  | Can I be cared at home if I deteriorate (SW) |
| **13.** | **17.** | What support is available to me if I choose to be cared for at home? |
| **14.** | **18.** | Who can help to decide about my care? |
| **15.** |  | Who will be responsible for my care if I deteriorate? (SW) |
|  | **19.** | Who will be my mediator if I deteriorate? (NL) |
|  | **20.** | What can I expect from the hospital regarding my treatment and what is the role of the general practitioner? (NL) |
|  | **21.** | What support is there at home in case of acute breathlessness (NL) |
| **SW** | **NL** | **Section 3: End-of-life** |
| **16.** |  | How will I know if I am approaching end-of-life (SW) |
|  | **22.** | Who will tell me when I am approaching the end-of-life? (NL) |
|  | **23.** | Will the last phase of my life be long? (NL) |
| **17.** | **24.** | What will happen to my heart failure treatment at the end-of-life? |
| **18.** |  | Living with heart failure, how will the end-of-life be? (SW) |
| **19.** |  | How will the last days in my life be, dying from heart failure, is there much suffering for example breathing problems and anxiety? (SW) |
|  | **25.** | What symptoms can occur in the last phase of my life? (NL) |
|  | **26.** | How can I record my preferred treatment wishes so they are known by my health care providers? (NL) |
| **SW** | **NL** | **Section 4. Other questions that family members may want to discuss** |
| **20.** |  | Are there things the person who is ill should avoid doing? (SW) |
|  | **27.** | What can I do if the person who is ill, is deteriorating and needs more care? (NL) |
| **21.** | **28.** | How do I best help my family member with heart failure if he/she deteriorates? |
| **22.** |  | If needed, how do I get help to look after my family member with heart failure? (SW) |
| **23.** |  | Who can I talk to about things that worry or bother me about the care given to my family member with heart failure? (SW) |
|  | **29.** | Who can I talk to if I am worried about the care given to my family member with heart failure? (NL) |
|  | **30.** | Who can I talk to about things that worry or bother me? (NL) |
| **24.** | **31.** | What support is there for me if my family member with heart failure deteriorates and I feel that I cannot do anymore? |
| **25.** | **32.** | How do I know that end-of-life is approaching? |
| **26.** |  | How will my family member with heart failure react when the illness deteriorates? (SW) |
|  | **33.** | How do patients in general react when the illness deteriorates? (NL) |
| **27.** |  | How will family members react when the family members with heart failure deteriorates? (SW) |
| **28.** |  | How do I know that my family member with heart failure has passed away? (SW) |
|  |  | **Section 5. Questions for patients with a heart failure pacemaker or an implantable cardioverter defibrillator** |
| **29.** | **34.** | What impact will my heart failure pacemaker (CRT), pacemaker or implantable cardioverter defibrillator (ICD) will have on my last days of life? (NL) |
| **30.** | **35.** | What will happen to my ICD/CRT/pacemaker treatment at the end-of-life? |
| **31.** | **36.** | How will the shock therapy of my ICD be switched off? |
| **32.** |  | What will happen to my heart if my ICD is switched off? (SW) |
|  | **37.** | What will happen if my ICD is switched off (NL) |
| **33.** | **38.** | Can the shock therapy of my ICD be switched off without mine or my family members’ knowledge? |
