# Supplementary material for: Optimizing of a question prompt list to improve communication about the heart failure trajectory in patients, families, and health care professionals
Source: BMC Palliat Care. 2020 Oct 15;19:161. doi: 10.1186/s12904-020-00665-3 (PMC7566035; doi:10.1186/s12904-020-00665-3)
Supplement: Supplementary file 2 — Additional file 2. The questions that were suggested to be deleted or added in the Swedish and Dutch Delphi rounds. SW, Sweden, NL, The Netherlands. [file 12904_2020_665_MOESM2_ESM.docx]

**Additional file 2.** The questions that were suggested to be deleted or added in the Swedish and Dutch Delphi rounds. SW, Sweden, NL, The Netherlands.

| **SW** | **NL** | **Questions found not relevant in round 1, suggested to be deleted from the QPL:** |
| --- | --- | --- |
| **14.** | **14.** | When I deteriorate, what support is there to be cared for at home? |
| **15.** |  | What help can I get if I choose to be cared for at home? |
| **19.** | **19.** | Is it possible to predict how long someone has left to live? |
| **20.** |  | Will the last phase of my life be long? |
| **21.** | **21.** | How will I feel during my last days of life? |
| **23.** | **23.** | What happens in the body when you die from heart failure? |
| **24.** |  | Are breathing problems common at the end of life? |
| **25.** | **25.** | Is pain common at the end of life? |
| **26.** | **26.** | Is it common to experience anxiety at the end of life? |
| **27.** | **27.** | How do we agree on what the person who is ill can/should/is allowed to do? |
| **34.** | **34.** | What do I reply to the question ”Am I going to die now?”? |
|  | **37.** | How do I know if the patient has died? |
| **38.** | **38.** | What happens with the dead body? |
|  | **39.** | Who can help me organise the funeral? |
|  | **40.** | What will happen to my ICD/CRT/pacemaker treatment at the end-of-life? |
|  |  | **Questions found missing in round 1, suggested to be added in the final QPL:** |
|  | **46.** | What can heart failure mean for my work or for the work of my partner? |
| **46.** |  | How do I keep my dignity when I am dying? |
|  | **47.** | What can heart failure mean for the relationship with my partner? |
| **47.** |  | How can the last phase of life be when you suffer from heart failure? |
|  | **48.** | Which phases of heart failure are there and in which phase am I? |
| **48.** |  | How will the last days in my life be, dying from heart failure, is there much suffering for example breathing problems and anxiety? |
|  | **49.** | How is the course of the disease in most of the patients with heart failure? |
| **49.** |  | Are there things the person who is ill should avoid doing? |
|  | **50.** | Will there be treatments in a foreseeable future that might be successful in curing heart failure? |
|  | **51.** | What can I expect from the hospital regarding the treatment and what is the role of the general practitioner? |
|  | **52.** | How can I best record my preferred treatment wishes, so that they are known by all healthcare professionals? |
|  | **53.** | With whom can I talk when I have problems with coping with the disease? |
|  | **54.** | What requirements are needed in my home in order for me to stay there? |
|  | **55.** | Can I stay at home in the last phase of my life? |
|  | **56.** | What support is there at home in case of acute breathlessness? |
|  | **57.** | Will I die immediately when my ICD / pacemaker is switched off? |
|  | **58.** | What is known about the influence of sexual activity on wellbeing and the course of the disease in patients with heart failure? |
|  | **59.** | Is the risk of other diseases (for example colon cancer) higher in patients with heart failure? |
